# The role of (voluntary) large language model use and individual needs in shaping work design

**DOI:** 10.1177/10519815261476647

**Published:** 2026-08-21

**Authors:** Valeria Maisch, Tanja Bipp, Marvin Walczok

**Affiliations:** 1 Department of Psychology, 9144 Heidelberg University, Heidelberg, Germany; 2 Department of Psychology, Ludwig-Maximilians-Universität (LMU) Munich, Munich, Germany

**Keywords:** large language models, voluntary use, work design, autonomy, information processing, role overload, individual needs, decision-making performance

## Abstract

**Background:**

Large Language Models (LLMs) are increasingly adopted in organizational practice to support task execution. However, their impact on work design, and ultimately on employee outcomes, remains unclear.

**Objective:**

Drawing on theoretical frameworks regarding the effects of technologies on work design, we investigate potentially negative effects of LLM use on three work characteristics (decision-making autonomy, information processing, role overload) and on decision-making performance. We examine the mitigating role of voluntary LLM use and the needs for autonomy and cognition as moderating factors.

**Methods:**

We conducted an online-experiment with 505 German workers from various industries, who performed two work tasks and were randomly assigned to one of three groups: no LLM use, instructed LLM use, or voluntary LLM use. Participants rated perceived work characteristics after task completion. Individual need strengths and decision-making performance were assessed.

**Results:**

Findings support that LLM use reduces decision-making autonomy, information processing, and decision-making performance, but also decreases role overload. We found support for the mitigating role of voluntary LLM use and a moderating effect of need for autonomy, amplifying the effects on decision-making autonomy. Results did not support moderating effects for need for cognition.

**Conclusions:**

Overall, our experiment provides causal evidence that voluntary LLM use mitigates the negative effects on work characteristics, such as reduced decision-making autonomy and information processing, and losses in decision-making performance while reaping the benefits, such as reduced role overload. Voluntary use seems to be particularly important for workers with a high need for autonomy.

## Introduction

*Large language models* (LLMs) – such as OpenAI's ChatGPT or Microsoft's Copilot - are increasingly adopted in organizational processes to support employees in task execution across various industries and occupations.^1,2^ Given their versatility and widespread adoption, researchers agree that LLMs will significantly influence work design.^1,3^ Defined as “the content and organization of one's work tasks, activities, relationships, and responsibilities”^4[p. 662]^, work design fundamentally affects key outcomes such as employee health, motivation, and performance.^5,6^ Bienefeld and Grote^7^ outline that AI-induced changes can enhance but can also decrease job quality, emphasizing the need for further research investigating AI's effects on work characteristics. However, an overarching model that can explain or predict the effects of such new technologies on work characteristics, potential moderating factors, and their consequences for work outcomes is currently lacking.^8,9^ Initial empirical evidence demonstrates particularly negative effects of LLM use, for example, for *decision-making autonomy* and *information processing*.^3^ Investigating the effects of LLM use on work characteristics and moderating factors on the individual and organizational level is essential to mitigate negative impacts on work design and, ultimately, on employee outcomes.

Therefore, we investigate the potentially negative effects of LLM use on three work characteristics and examine the conditions under which these effects might be mitigated or vary across individuals. Our study addresses the following four research questions: First, does LLM use have negative impacts on work characteristics in the form of reduced perceptions of decision-making autonomy and information processing or increased perceptions of role overload? Second, does voluntary LLM use mitigate the potentially negative effects of LLM use on work characteristics? Third, does individual need strength – in terms of need for autonomy or cognition – moderate the effects of LLM use on work characteristics? Fourth, does (voluntary) LLM use affect decision-making performance?

For this, we administered an online experiment with German employees (*N* = 505) from various industries, in which participants were randomly assigned to complete two work tasks in one of three conditions: either without LLM use, with explicit instruction to use an LLM, or with the option of voluntary LLM use. Therefore, our experiment provides causal evidence for the effects of LLM use on work characteristics and sheds light on the theoretically proposed (e.g.,^10^) yet empirically untested moderating effects of higher-level (voluntary use) and individual factors (need strength) to achieve benefits of digital technologies in terms of work design. By integrating voluntariness of LLM use as well as individual needs as moderating factors – and by including role overload as a largely unexplored work characteristic – we make an essential contribution to the development of comprehensive theories and models that outline how LLM use impacts work design. Through a better understanding of these effects, we seek to offer practical guidance for the implementation of LLMs in organizational practice, promoting beneficial work design in today's world of work.

## Theoretical background

### Large language models (LLMs)

*Generative AI* – defined as “computational techniques that are capable of generating seemingly new, meaningful content such as text, images, or audio from training data”^2[p. 111]^ – represents the technical foundation for generative AI systems including LLMs. LLMs refer to neural networks that model and generate text data using “large-scale, sequential neural network[s]”^2[p. 114]^.They are pre-trained on auxiliary tasks and massive text corpora, and fine-tuned using task-specific datasets. LLMs enable the generation of multimodal outputs in natural language that can be used for a wide range of work tasks, from managerial decision-making tasks to creative and artistic tasks. Employees across different occupations use LLMs for various purposes, including accelerating workflows, generating summaries for professional support, or generating graphics and videos using text input. Due to rapid advancements in LLM technology, these models increasingly have the potential to transform even complex work tasks across industries, such as medical and legal consulting or code generation.^2^ However, meta-analytic evidence suggests that working with AI does not per se lead to performance gains,^11^ emphasizing the need to understand how LLM use alters work design in order to support the benefits of working with AI.

### LLM use and work design

Despite the importance of work design for various employee outcomes,^5,6^ no overarching theory yet exists to describe and explain how technologies such as LLMs affect specific work characteristics.^8,9^ However, several theoretical frameworks and reviews emphasize that technology integration impacts work design in various ways, for example, how employees perceive autonomy at work.^10,12^ For our investigation of the effects of LLM use at work, we build on a central conclusion Parker and Grote^10^ drew from their literature review. They outline that technology has no predetermined effect on work design, but that such effects depend on several factors: the type of technology itself (in our case, LLMs), higher-level factors (e.g., organizational factors), and individual factors (e.g., personality traits). Although researchers agree that these interacting factors influence work design,^10,12^ empirical investigations of underlying assumptions and their combined effects remain scarce.^9^

Therefore, we aim to explain in our study under which circumstances and for whom the effects of LLM use on work design might vary. First, building on empirical evidence indicating negative effects of LLM use on work characteristics,^1,3^ we investigate how the explicit instruction to use an LLM for work tasks impacts perceptions of decision-making autonomy, information processing, and role overload. Second, based on the important role of user control in avoiding undesired effects of AI use,^13^ we examine voluntariness of LLM use as an organizational factor that might mitigate these potentially negative effects. Third, we aim to delineate for whom such effects are more or less likely to occur and investigate the potential moderating role of two individual factors: need for autonomy and need for cognition. Fourth, we investigate the effects of (voluntary) LLM use on decision-making performance.

### LLM use and decision-making autonomy, information processing, and role overload

To investigate the effects of LLM use on work design, we focused on three work characteristics that are highly relevant for employee health, motivation, and performance,^5,6^ and for which negative effects have been indicated in the empirical literature on LLM use.^1,3^ Therefore, we focused on the impact of instructed LLM use – compared to non-use – on the perception of decision-making autonomy, information processing, and role overload. Given that, to our knowledge, there are only few experimental investigations regarding the effects of LLMs use on work design (e.g.,^3^), we build our expectations also on findings for other technologies.

First, decision-making autonomy refers to the degree of independence and freedom a job offers regarding work-related decisions and is one of three interrelated aspects of job autonomy.^14^ Bienefeld and Grote^7^ outline mixed implications of AI integration for job autonomy, thereby highlighting the importance of further investigations. While it has been argued that AI may increase job autonomy through automation of simple tasks,^7^ research indicates that increased automation reduces perceived autonomy.^10,15^ For example, working with intelligent assistance systems in assembly has been shown to reduce different autonomy facets, including decision-making autonomy,^8^ and interacting with chatbots for web search has been shown to reduce overall autonomy.^16^ Initial research on LLM use in particular indicates that it reduces perceived control and decision-making autonomy.^3,17^ To further examine mitigating factors of these negative effects regarding LLM use, we aim to corroborate these findings.

*H1:* Individuals who are instructed to work with an LLM perceive lower decision-making autonomy than individuals who complete the tasks without an LLM.

Second, information processing refers to „the degree to which a job requires attending to and processing data or other information“^14[p. 1323]^ and is cognitive by nature.^18^ While new technologies have the potential to reduce cognitive demands at work, empirical evidence for such effects seems mixed. On the one hand, studies show that working with intelligent assistance systems in assembly increases perceived information processing,^8,9^ whereas interacting with chatbots for web search increases cognitive load.^16^ On the other hand, Parent-Rocheleau and Parker's^18^ literature review attributes automation's negative effects on autonomy to reduced task involvement. This conclusion aligns with research demonstrating that working with AI enables cognitive offloading,^20^ thereby reducing cognitive effort expended on tasks.^21^ Consequently, AI use likely decreases task involvement through cognitive offloading, leading to reduced information processing. Indeed, empirical evidence from experimental studies demonstrates that AI-based decision support systems reduce information load^22^ and that LLM use specifically reduces perceived information processing.^3,17^ Given the predominance of this theoretical argument and empirical evidence, we hypothesize that LLM use leads to lower perceptions of information processing.

*H2:* Individuals who are instructed to work with an LLM perceive lower information processing requirements than individuals who complete the tasks without an LLM.

Third, role overload “refers to employees perceiving that they have excess responsibilities given their time and capability constraints”.^18[p. 272]^ The integration of AI in the workplace may have dual implications for role overload. On the one hand, increased automation has been shown to reduce workload^23^ and AI seems to be perceived as a helpful resource rather than a (hindering) demand by employees.^24^ Empirical evidence further suggests that LLM use increases efficiency in text generation and reduces perceived information processing,^3,17^ which should also reduce role overload. On the other hand, digitalization and AI adoption can increase overload and demands,^1,25^ as LLM use in particular creates new tasks such as prompt engineering and output adaptation.^26^ Moreover, LLM integration may lead to task complexity polarization,^26^ simplifying easier tasks while increasing the complexity of challenging tasks. These conflicting expectations preclude clear predictions about the effects of instructed LLM use on role overload. Therefore, we explore whether individuals differ in terms of perceived role overload if they are instructed to work with an LLM compared to those who complete the tasks without an LLM (Exploratory research question (ERQ) 1).

### Mitigating effects of voluntary LLM use

Drawing from the assumption that the effects of technology on work design depend on higher-level (e.g., organizational) factors^10^ and emphasizing the importance of human control in AI integration,^13,27^ we investigate if voluntariness of LLM use mitigates the negative effects of instructed LLM use. Having the freedom to voluntarily choose whether to work with LLMs enhances human control and autonomy, particularly in terms of work methods autonomy – reflecting the freedom to choose methods for task execution.^14^

Parent-Rocheleau and Parker^19^ suggest in their literature review that negative effects of workplace automation may be attributed to reduced involvement in work tasks and may be mitigated by greater control over the system. Especially when work tasks involve complex decision-making or variations in workload, flexible approaches that grant human control may be more adequate.^15^ Particularly in AI-assisted work, voluntary use is emphasized as a key factor in mitigating negative effects on autonomy and motivation.^28^ Taken together, voluntariness of LLM use should enhance task involvement and provide greater control over workload variations. Therefore, we expect voluntariness of LLM use to act as a mitigating factor regarding the potential negative effects of instructed LLM use on perceived decision-making autonomy, information processing, and role overload.

First, as voluntariness of LLM use increases work-methods autonomy compared to instructed LLM use or non-use, decision-making autonomy should also increase, given the theoretical and empirical interrelations among different autonomy facets.^14^ Though theoretically postulated,^27,28^ empirical findings regarding the effects of voluntary LLM use on autonomy are still lacking. Voluntariness of LLM use can be compared to adaptable automation – a form of flexible automation that keeps decision-making power over task allocation within human control – which has been shown to increase perceived autonomy compared to static decision automation.^15^ Given that voluntarily choosing whether to use an LLM grants additional decision-making freedom even compared to no LLM use, we expect voluntary LLM use to increase perceived decision-making autonomy compared to both instructed LLM use and non-use.

*H3:* Individuals who can choose to work voluntarily with an LLM perceive higher decision-making autonomy than (a) individuals who are instructed to work with an LLM and (b) individuals who complete the tasks without an LLM.

Second, working with AI can facilitate cognitive offloading,^20^ thereby reducing the cognitive resources allocated to tasks^21^ and consequently resulting in reduced task involvement. However, adaptable automation has been shown to increase active involvement compared to static decision automation.^15^ Therefore, active involvement should also be higher with voluntary LLM use compared to instructed LLM use – resulting in higher perceived information processing.

*H4:* Individuals who can choose to work voluntarily with an LLM perceive higher information processing requirements than individuals who are instructed to work with an LLM.

In the literature on the potentially mitigating effects of increased control in technology implementation, more flexible approaches are mostly compared to static approaches (e.g.,^15^), but not to work situations without technology use. Consequently, there is – to our knowledge – no prior empirical evidence to predict the differences between voluntary LLM use and non-use in terms of information processing. Voluntary LLM use could either partially or fully mitigate the reduction of perceived information processing. Therefore, we explore if individuals differ in terms of perceived information processing requirements if they can choose to work voluntarily with an LLM compared to those who complete the tasks without an LLM (ERQ 2).

Third, considering the phenomenon of task-complexity polarization^26^ and the risk of workload variations,^10^ voluntariness of LLM use provides potential benefits regarding role overload through granting the freedom of working with LLMs only when reducing workload. Comparably, adaptable automation has been shown to reduce workload compared to no automation as well as low or intermediate levels of automation.^29,30^ Transferring these effects to LLM use, voluntariness should result in lower perceived role overload than instructed LLM use and non-use.

*H5:* Individuals who can choose to work voluntarily with an LLM perceive lower role overload than (a) individuals who are instructed to work with an LLM and (b) individuals who complete the tasks without an LLM.

### Moderating effects of individual need strength

Building on the assumption that the effects of technology on work design also depend on individual factors,^10^ we propose that need for autonomy moderates the effects of LLM use on perceived decision-making autonomy, while need for cognition moderates the effects on perceived information processing. First, need for autonomy describes an individual's desire to experience a sense of choice and psychological freedom in their activities.^31,32,33^ Based on the principle of congruent fit between personal and organizational characteristics,^34^ a work situation emphasizing autonomy might be more beneficial for employees with a high need for autonomy. Research on blended work supports this assumption, which is – due to its increased autonomy – perceived as more effective by individuals with a high need for autonomy.^33^ Additionally, individuals with a strong dispositional orientation towards autonomy have shown larger benefits from an autonomy supportive environment.^35^ Taken together, we expect that individuals with a higher need for autonomy perceive instructed LLM use – compared to no LLM use – as particularly restrictive, and voluntary LLM use as particularly liberating.

*H6:* The need for autonomy moderates the effects of (a) instructed LLM use – as described in H1 – and (b) voluntary LLM use – as described in H3 – on perceived decision-making autonomy, such that the effects are stronger for individuals with a higher need for autonomy.

Second, need for cognition describes an individual disposition to enjoy and engage in effortful cognitive activities.^36^ Due to its ease of use, AI facilitates faster, less elaborate thinking and enables cognitive offloading,^20^ thereby allowing individuals to delegate cognitively demanding tasks to AI. While research investigating individual needs in relation to cognitive offloading appears to be lacking,^37^ the effects of perceived ease of use on technology adoption intentions have been shown to be stronger among individuals with a low need for cognition.^38^ Therefore, individuals with a low need for cognition should be more prone to offload cognitive effort to an LLM and perceive less information processing. In addition, individuals with a high need for cognition have been shown to perform better when solving problems with AI, suggesting that intrinsic motivation to engage in cognitive tasks remains high even when AI is available.^39^ Therefore, individuals with a high need for cognition should be more prone to use an LLM to stimulate and expand cognition, thereby perceiving more information processing.

*H7:* The need for cognition moderates the effect of instructed LLM use – compared to no LLM use – on perceived information processing requirements, such that (a) the effect is negative for individuals with a low need for cognition, and (b) positive for individuals with a high need for cognition.

### Decision-making performance

Building on the role of work design for key employee outcomes,^5,6^ we investigate how (voluntary) LLM use affects performance in a decision-making task. In their conceptual model linking generative AI use to job performance, Hickman and Langer^27^ propose mandatory use as a moderator of work characteristics’ effects on performance, emphasizing the importance of freedom of choice when using generative AI. These authors argue that mandatory AI use reduces perceived autonomy, thereby reducing motivation and job performance. Building on this assumption, voluntary LLM use should enhance job performance compared to both no LLM use and instructed use. This expectation aligns with automation research showing that adaptable automation improves performance compared to no automation and low or intermediate levels of static automation.^29,30^ Given that human-AI collaboration seems improvable for decision-making tasks,^11^ we focused on performance in a decision-making task that targets work design,^40^ operationalized through the number of enriching tasks participants selected.

*H8:* Individuals who can choose to work voluntarily with an LLM allocate more enriching tasks than (a) individuals who are instructed to work with an LLM and (b) individuals who complete the tasks without an LLM.

Turning to the comparison of instructed and no LLM use, theoretical arguments and empirical evidence appear inconclusive. While AI use can enhance decision-making by leveraging big data and machine learning,^10^ mandatory use may impair performance by undermining autonomy.^27^ Therefore, we explore whether individuals differ in terms of enriching task allocation if they are instructed to work with an LLM compared to those who complete the tasks without an LLM (ERQ3).

## Method

To test our hypotheses, we conducted an online experiment, in which we simulated work tasks in an HR context and participants were randomly assigned to one of three groups: task completion without LLM use, with explicit instruction to use an LLM, or with the option of voluntary LLM use. As ChatGPT is the most widely used LLM in Germany,^41^ we implemented ChatGPT in our experiment.

### Open science

We pre-registered all measures and hypotheses before data collection (https://osf.io/r6xz2).

### Experimental design and procedure

Participants first completed self-report measures on individual needs. Second, they were randomly assigned to one of three experimental groups to work on two tasks: task completion without LLM use (instruction group 1: “Please complete these tasks without any aids”), task completion with the explicit instruction to work with an LLM (group 2: “Please complete these tasks using ChatGPT”), and task completion with voluntary LLM use (group 3: “You are free to decide whether to use ChatGPT or to complete the tasks without any aids”). In group 2 and 3, participants first received a standardized introduction explaining ChatGPT's core functionalities. For them, access to ChatGPT (model: gpt-5-nano) was provided through a proxy-secured interface to safeguard participant privacy. After completing both tasks, all participants were asked to rate work characteristics during task completion (presented in randomized order) and provided demographic information (e.g., age, sex, and prior LLM experience). An attention check (“Please select *strongly agree* (5) here”) was included to identify inattentive responding. To ensure careful task engagement, we implemented a mandatory short delay on all task pages, preventing participants from proceeding too quickly. At the end of the study, we asked participants if they had used ChatGPT (or another LLM) to complete either of the two tasks. Participants in the no LLM condition were excluded for reporting LLM use, while participants in the instructed LLM condition were excluded for not using an LLM.

### Tasks

All participants completed two HR-related tasks, selected for their relevance to decision-making and text generation as core competences of LLMs. First, participants completed a decision-making task targeting work design,^40^ assigning four additional tasks from a list of 11 to a new job position, which already included four predefined tasks. Second, participants drafted a job advertisement for the position created in the first task (all individually selected tasks were displayed for this), targeting a fictitious company. Instructions required them to design an appealing advertisement covering core key aspects: job tasks (e.g., a section titled “Your Responsibilities”), qualifications (e.g., “Your Profile”), and employee benefits (e.g., “We Offer”).

### Participants

We recruited German employees across diverse industries through personal and professional networks to participate in our online experiment conducted between December 2025 and February 2026. Additionally, part-time students from a professional study program were invited to participate in exchange for course credits. Eligibility criteria required participants to be at least 18 years old and currently employed for a minimum of 10 h per week. We offered three incentives for participation (summary of study results, feedback on individual needs scores, and a raffle of five 20€ vouchers).

A total of 618 individuals completed the survey. Several participants had to be excluded from the analysis. First, five participants had to be excluded because they actively declined data processing at the end of the survey: after survey completion, participants were debriefed about the LLM manipulation and asked to reconfirm their initial consent for study participation. Second, 54 participants were excluded based on our eligibility criteria: one participant failed to indicate their age, five participants reported less than 10 working hours per week, and 48 participants did not pass the attention check. Third, 30 participants in the no LLM condition were excluded for reporting LLM use, while 17 participants in the instructed LLM condition were excluded for not using an LLM. Forth, seven participants were excluded due to job advertisements that failed to meet predefined criteria (covering at least two of the three key aspects included in the instructions), suggesting inattentive task completion. Finally, data was excluded from the analysis if more than 50% of the items per scale were missing.

The final sample comprised *N* = 505 participants. Women were overrepresented (67%) compared to men (32%); two participants (1%) identified as non-binary. Participants’ age ranged from 18 to 64 (*M* = 29.26, *SD* = 9.79) years. The sample was predominantly academic, with 42% holding a university degree, while 36% reported vocational training as their highest qualification. Occupational affiliation was assessed across 16 occupational sectors and an additional “other” category. The most represented occupational sector was business, management, and administration (15%), followed by finance (13%), marketing and sales (11%), and healthcare (7%). Overall, the sample reflected a broad range of occupational backgrounds, with participants representing all major occupational sectors. On average, participants worked 34.45 h per week (*SD* = 10.12), with 80% in office-based roles.

### Manipulation check: voluntary use

To verify the successful manipulation of voluntary LLM use, participants responded to three items assessing work methods autonomy^14^^[p. 1323]^. We used a validated German version^42^ of the Work Design Questionnaire (WDQ^14^) with items modified to reference the two tasks completed in the study (example: “I was allowed to make decisions about what methods I use to complete the tasks.”, α = .84). Responses were collected on a 5-point Likert scale ranging from *strongly disagree* (1) to *strongly agree* (5). Work methods autonomy was significantly higher with voluntary use (*M* = 3.79, *SD* = .82) than with no LLM use (*M* = 3.15, *SD* = 1.02, *t_Welch_*(290.1) = 6.20, *p* < .001, *d* = .70) or instructed use (*M* = 2.48, *SD* = 1.02, *t_Welch_*(336.68) = 13.35, *p* < .001, *d* = 1.42), indicating the successful manipulation of voluntariness.

### Measures

All items assessing work characteristics were slightly adapted to reference to the two experimental tasks (e.g., replacing “the job” with “the tasks”). Unless otherwise specified, responses were collected using a 5-point Likert scale from *strongly disagree* (1) to *strongly agree* (5).

#### Work characteristics

**Decision-making autonomy.** We used the 3-items scale assessing decision-making autonomy (“The tasks allowed me to make a lot of decisions on my own”, α = .89) from the validated German version^42^ of the WDQ.^14^

**Information processing.** We used the 4-items scale assessing information processing (“The tasks required me to monitor a great deal of information”, α = .83) from the validated German version^42^ of the WDQ.^14^

**Role overload**. Comparable to Parker and Knight,^18^ we assessed role overload using three items from the pace and amount of work subscale (“I had too much work to do”, α = .73) of the German version of the Questionnaire on the Experience and Evaluation of Work 2.0.^43^

#### Individual needs

**Need for autonomy.** We used a translated version of the 4-item scale developed by Van Yperen et al.^33^ to measure work-related need for autonomy (“At work I have the need to determine on my own how to best approach my work”, α = .88). Responses were collected on a 7-point Likert scale ranging from *not at all* (1) to *to an extremely large extent* (7).

**Need for cognition**. We used the German translation^44^ of the 6-item scale (NCS-6) from Lins De Holanda Coelho et al.^45^ to measure need for cognition (“I would prefer complex to simple problems.”, α = .82). Responses were collected on a 7-point Likert scale from *completely inaccurate* (1) to *completely accurate* (7).

#### Performance

**Enriching task allocation**. Performance in the first work task was operationalized by counting the number of enriching tasks selected (range: 0–4). The task, originally developed by Parker et al.,^40^ was translated into German and had been successfully used in a pilot study.^46^

#### Prior LLM experience

Participants’ experience with LLMs was assessed using two self-developed items measuring prior exposure (dichotomous: *yes* / *no*) and its extent on a 5-point Likert scale. We compared prior experience across the two LLM groups. Results revealed that only 10 participants (3%) reported no prior experience, and the extent of experience did not differ significantly between the two groups (*t*(349*)* = 1.12, *p* = .262, *d* = .12). Given this lack of meaningful variation, prior experience was excluded as a variable in further analyses.

### Analyses

For H1 - 5 and H8, we conducted one-way ANOVAs to assess group differences, followed by planned contrasts for pairwise group comparisons. For H6 and H7, we performed regression analyses using needs, group contrasts, and their interactions as predictors. The significance level was set at α = .05 for hypothesis testing and adjusted to α = .01 for exploratory analyses.

## Results

### Descriptive statistics

Table 1 presents the means, standard deviations, internal consistencies (Cronbach's α), and bivariate correlations among the study variables. All scales demonstrated good to satisfactory internal consistency.

**Table 1.** Means, standard deviations, internal consistencies, and correlations among the study variables (N = 505).

Variables	Range	*M*	*SD*	1	2	3	4	5	6	7
1. Decision-making Autonomy	1–5	3.24	1.05	(.89)						
2. Information Processing	1–5	2.46	.81	.32***	(.83)					
3. Role Overload	1–5	1.97	.83	.18***	.45***	(.73)				
4. Work Methods Autonomy	1–5	3.14	1.10	.78***	.25***	.12**	(.84)			
5. Need for Autonomy	1–7	5.75	.93	−.05	−.07	−.14**	−.04	(.88)		
6. Need for Cognition	1–7	4.92	1.01	−.11*	−.11*	−.14**	−.09*	.32***	(.82)	
7. Enriching Task Allocation	0–4	2.51	1.58	.14**	.01	.05	.10*	.08	.14**	-^a^

*Notes.* Bivariate Spearman intercorrelations due to deviations from normality for Need for Autonomy. *N* = 504 for Need for Autonomy. Cronbach’s α in brackets. ^a^Internal consistency could not be assessed for enriching task allocation due to its single-item measurement. *p < .05. **p < .01. ***p < .001.

### Perceptions of work characteristics

To examine differences in perceived work characteristics across the three experimental groups, we conducted a series of one-way ANOVAs. Table 2 presents the cell means and standard deviations for each group, and ANOVA results, revealing significant differences between our three experimental groups for decision-making autonomy (*F*(2, 502) = 41.02, *p* < .001, *η_p_^2^* = .14), information processing (*F*(2, 502) = 9.73, *p* < .001, *η_p_^2^* = .04), and role overload (*F*(2, 502) = 28.80, *p* < .001, *η_p_^2^* = .10). Follow-up planned contrasts were performed to compare the groups pairwise for each characteristic to test our hypotheses (Table 3).

**Table 2.** Cell means and standard deviations of the study variables across the experimental conditions and ANOVA results for the dependent variables.

		(1) no LLM use		(2) instructed LLM use		(3) voluntary LLM use		
		*n* = 153		*n* = 178		*n* = 174		
	Range	*M*	*SD*	*M*	*SD*	*M*	*SD*	One-Way ANOVA Results
Decision-making Autonomy	1–5	3.45	.98	2.72	1.03	3.60	.90	*F*(2, 502) = 41.02, *p* < .001, *η_p_^2^* = .14 ***
Information Processing	1–5	2.67	.71	2.28	.86	2.47	.80	*F*(2, 502) = 9.73, *p* < .001, *η_p_^2^* = .04 ***
Role Overload	1–5	2.36	.82	1.72	.74	1.87	.81	*F*(2, 502) = 28.80, *p* < .001, *η_p_^2^* = .10 ***
Enriching Task Allocation	0–4	2.67	1.58	2.20	1.63	2.68	1.49	*F*(2, 502) = 5.26, *p* = .006, *η_p_^2^* = .02 **
Need for Autonomy	1–7	5.72	.99	5.81	.94	5.72	.85	
Need for Cognition	1–7	4.87	1.03	4.96	1.03	4.92	.98	

*Notes. n* = 173 for Need for Autonomy in Group 3: Voluntary LLM Use. *p < .05. **p < .01. ***p < .001.

**Table 3.** Results of planned contrasts for the dependent variables (work characteristics and decision-making performance).

	Group 2–1 (Instructed – no LLM use)	Group 3–2 (Voluntary – instructed LLM use)	Group 3–1 (Voluntary – no LLM use)
Decision-making Autonomy	**H1:** *t*(502) = -6.84, *p* < .001, *d* = .76 ***	**H3a:** *t*(502) = 8.48, *p* < .001, *d* = .91 ***	**H3b:** *t*(502) = 1.35, *p* = .176, *d* = .15
Information Processing	**H2:** *t*(502) = -4.41, *p* < .001, *d* = .49 ***	**H4:** *t*(502) = 2.20, *p* = .028, *d* = .24 *	**ERQ2:** *t*(502) = -2.27, *p* = .024, *d* = .25
Role Overload	**ERQ1:** *t*(502) = -7.34, *p* < .001, *d* = .81 **	**H5a:** *t*(502) = 1.81, *p* = .070, *d* = .19	**H5b:** *t*(502) = -5.56, *p* < .001, *d* = .62 ***
Enriching Task Allocation	**ERQ3:** *t*(502) = -2.72, *p* = .007, *d* = .30 *	**H8a:** *t*(502) = 3.85, *p* = .005, *d* = .30 **	**H8b:** *t*(502) = 0.03, *p* = .977, *d* = .003

*Notes.* For hypotheses (H): *p < .05. **p < .01. ***p < .001. For exploratory research questions (ERQ) an adapted significance level α = .01 was used: *p < .01. **p < .001.

#### Decision-making autonomy

As predicted in H1, decision-making autonomy was significantly lower with instructed LLM use than non-use, *t*(502) = -6.84, *p* < .001, *d* = .76. H3 received partial support: decision-making autonomy was highest with voluntary LLM use and significantly higher than with instructed use, *t*(502) = 8.48, *p* < .001, *d* = .91, but not significantly higher than with non-use, *t*(502) = 1.35, *p* = .176, *d* = .15.

#### Information processing

As postulated in H2, information processing was significantly lower with instructed LLM use than non-use, *t*(502) = -4.41, *p* < .001, *d* = .49. Consistent with H4, information processing was significantly higher with voluntary LLM use than instructed use, *t*(502) = 2.20, *p* = .028, *d* = .24. Regarding ERQ 2, information processing was higher with no LLM use than voluntary use, but this difference was not significant at the adjusted level of α = .01, *t*(502) = -2.27, *p* = .024, *d* = .25.

#### Role overload

Regarding ERQ 1, role overload was significantly higher with no LLM use than instructed use, *t*(502) = -7.34, *p* < .001, *d* = .81. H5 was partially supported: role overload was significantly lower with voluntary LLM use than non-use, *t*(502) = -5.56, *p* < .001, *d* = .62, but not significantly lower than with instructed use, *t*(502) = 1.81, *p* = .070, *d* = .19.

### Moderating effects of individual needs

To test the proposed moderating effects of need for autonomy and need for cognition, we performed regression analyses using needs, group contrasts, and their interactions as predictors (Table 4).

**Table 4.** Outcomes of the moderated regression analyses for variables predicting work characteristics.

	Decision-making autonomy						Information processing	
	Model 1 (*n* = 331)		Model 2 (*n* = 351)		Model 3 (*n* = 326)		Model 4 (*n* = 331)	
Variables	*B(SE)*	β	*B(SE)*	β	*B(SE)*	β	*B(SE)*	β
Need for Autonomy	.12 (.08)	.12	−.25 (.07)	−.24***	.12 (.07)	.12		
Need for Cognition							−.09 (.06)	−.11
Contrast 1^a^: Instructed – No LLM Use	−.72 (.11)	−.35***					−.38 (.09)	−.24***
Contrast 2^b^: Voluntary – Instructed LLM Use			.86 (.10)	.41***				
Contrast 3^c^: Voluntary – No LLM Use					.14 (.10)	.07		
Contrast 1^a^ x Need for Autonomy	−.37 (.11)	−.26***						
Contrast 2^b^ x Need for Autonomy			.26 (.11)	.16*				
Contrast 3^c^ x Need for Autonomy					−.11 (.11)	.07		
Contrast 1^a^ x Need for Cognition							.00 (.09)	.00
*F*	19.82		28.78		1.61		7.87	
df1, df2	3, 327		3, 347		3, 322		3, 327	
*Adjusted R* ^2^	.15***		.19***		.01		.06***	

*Notes.* ^a^Dummy: 1 = Instructed LLM Use, 0 = No LLM Use. ^b^Dummy: 1 = Voluntary LLM Use, 0 = Instructed LLM Use. ^c^Dummy: 1 = Voluntary LLM Use, 0 = No LLM Use. *p < .05. **p < .01. ***p < .001.

#### Need for autonomy

Regression analyses revealed that need for autonomy significantly moderated both significant group differences in decision-making autonomy: interactions with contrasts between instructed and no LLM use (β = −.26, *p* < .001) and voluntary and instructed LLM use (β = .16, *p* = .016) were significant predictors of decision-making autonomy. Figure 1 depicts these effects, indicating stronger effects for a higher need for autonomy. The interaction of need for autonomy with the group contrast between voluntary and no LLM use was not a significant predictor, β = .07, *p* = .286. Therefore, H6 received partial support.

**Figure 1.**
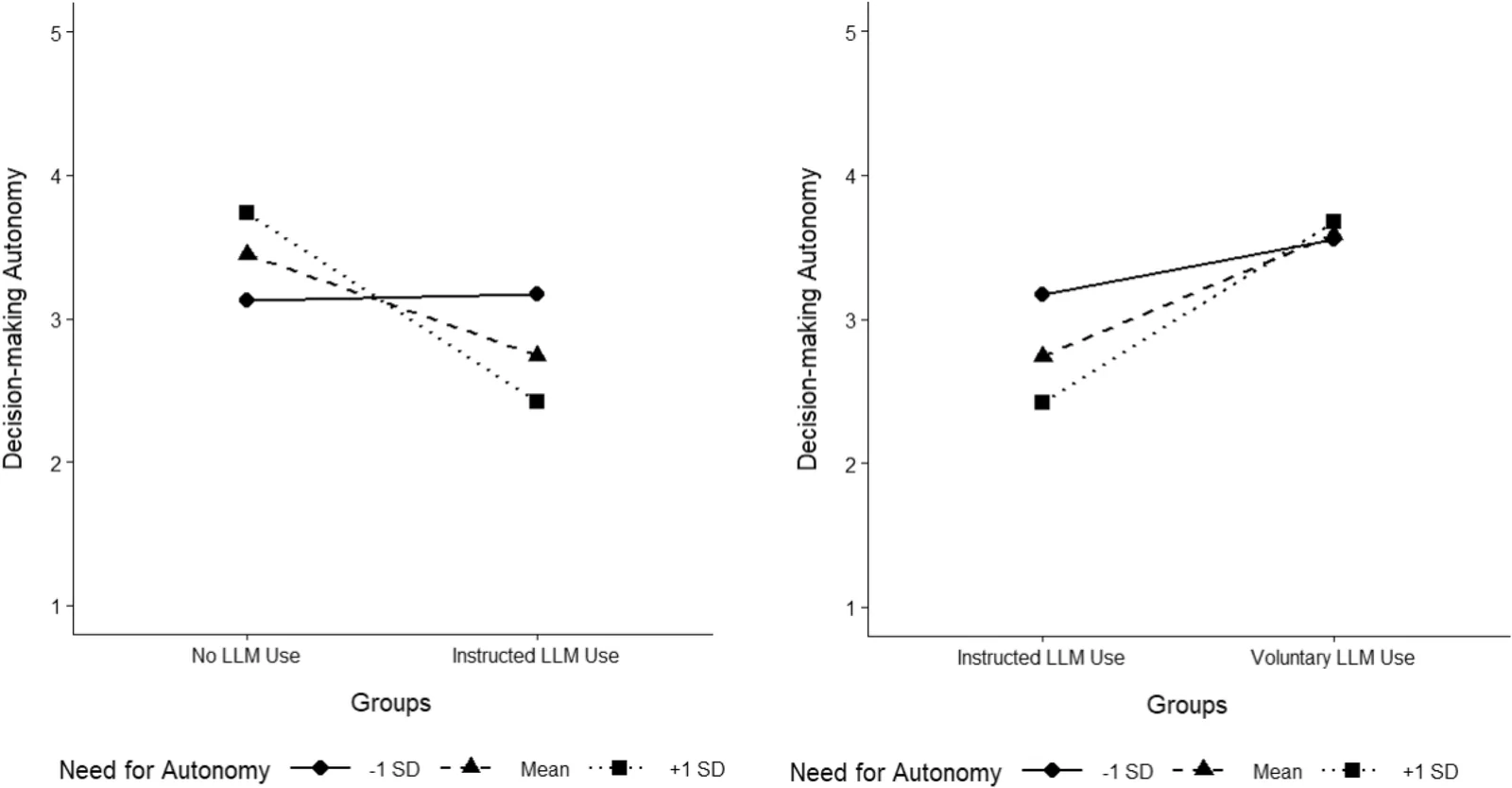
Mean decision-making Autonomy for Group Contrasts Moderated by Need for Autonomy.

#### Need for cognition

Regression analyses indicated that need for cognition did not significantly moderate the group differences in information processing between instructed and no LLM use: the interaction of need for cognition with the group contrast was not a significant predictor, β = .00, *p* = .994. Thus, H7 was not supported.

### Decision-making performance

To assess differences in decision-making performance, we conducted a one-way ANOVA (Table 2), which revealed significant group differences, *F*(2, 502) = 5.26, *p* = .006, *η_p_^2^* = .02. Therefore, we conducted planned contrasts for pairwise group comparisons (Table 3). H8 was partially supported: enriching task allocation was significantly higher with voluntary LLM use than instructed use, *t*(502) = 3.85, *p* = .005, *d* = .30, but did not differ significantly from non-use, *t*(502) = 0.03, *p* = .977, *d* = .003. Regarding ERQ 3, enriching task allocation was significantly higher with no LLM use than instructed use, *t*(502) = -2.72, *p* = .007, *d* = .30.

## Discussion

Building on Parker and Grote's^10^ theoretical framework, we provide causal evidence on the effects of LLM use on work design and, in addition, investigated the influence of organizational and individual factors on such effects. Overall, we found support for the majority of our hypotheses, by outlining negative effects of LLM use on perceived decision-making autonomy, perceived information processing, and decision-making performance. At the same time, we found a positive effect of LLM use in terms of decreasing perceptions of role overload. While voluntary use and need for autonomy had an impact on these effects, our results did not support a moderating role for need for cognition.

In detail, first, we examined how instructed LLM use affects perceived decision-making autonomy, information processing, and the – so far – understudied work characteristic of role overload. In line with initial empirical evidence,^3,17^ we found instructed LLM use (compared to non-use) to negatively impact perceived decision-making autonomy and information processing. Given conflicting expectations, we explored the effects of instructed LLM use on role overload and found a significant reduction compared to non-use. Collectively, these findings suggest that instructed LLM use diminishes core work characteristics with negative consequences for work design – but also reduces role overload, thereby potentially making work more tolerable.

Second, based on theoretical literature emphasizing the importance of human control in AI integration,^13,27^ we examined voluntary LLM use as a potential mitigating factor for the negative effects of instructed LLM use on work design. Supporting our hypotheses of its mitigating role, voluntary LLM use significantly increased perceived decision-making autonomy and information processing compared to instructed use. However, contrary to our hypothesis (H3b), decision-making autonomy was not significantly higher with voluntary than no LLM use, suggesting that voluntariness may not increase decision-making autonomy beyond non-use. Also, perceived information processing did not significantly differ between voluntary and no LLM use. Regarding role overload, voluntary LLM use led – as expected – to significantly lower levels than non-use, but – contrary to our expectations (H5a) – there was no significant difference between voluntary and instructed LLM use. These findings suggest that LLM use, whether voluntary or instructed, can reduce role overload compared to non-use.

Third, we investigated the moderating roles of need for autonomy and need for cognition in the effects of LLM use on work characteristics. We hypothesized that need for autonomy would amplify the effects of LLM use on perceived decision-making autonomy, with stronger impacts for individuals with a high need for autonomy. Indeed, in line with our predictions, we found that need for autonomy significantly moderates the effects of instructed LLM use (compared to non-use) and voluntary LLM use (compared to instructed use). However, need for autonomy did not affect the comparison between voluntary and no LLM use for decision making autonomy (as outlined in H6b). Nonetheless, our results suggest that individuals with a high need for autonomy perceive instructed LLM use as particularly restrictive and voluntary use as particularly liberating compared to individuals with a low need for autonomy. Contrary to our expectation (H7), need for cognition did not significantly moderate the effect of instructed LLM use (compared to non-use) on perceived information processing, indicating no relevant influence regarding LLMs’ effects on information processing.

Fourth, recognizing that human-AI collaboration in decision-making tasks seems improvable,^11^ we examined whether voluntary LLM use mitigates potential performance losses in a work design related decision-making task. Due to missing clear theoretical frameworks and limited prior empirical evidence, we explored performance differences between instructed and no LLM use. Interestingly, we found significant performance losses with instructed LLM use compared to non-use. Consistent with our expectations, voluntary LLM use led to performance gains compared to instructed use, again supporting its mitigating effect. However, contrary to H8b, there was no significant difference in decision-making performance between voluntary and no LLM use. This suggests that while voluntariness can improve decision-making performance relative to instructed use, it does not outperform working without LLMs.

### Theoretical implications

In alignment with the overview provided by Parker and Grote,^10^ we found causal evidence for effects of LLM use on work design, and support for the suggested role of higher-order (voluntariness) and individual (need strength) factors. Consistent with initial empirical evidence,^3,17^ we demonstrate negative effects of instructed LLM use on perceived decision-making autonomy and information processing, revealing adverse consequences for work design. However, we also found that LLM use seems to reduce role overload – a novel insight indicating positive consequences for work design. Notably, reductions in role overload may go hand in hand with lower levels of information processing and cognitive engagement, potentially diminishing essential elements of meaningful work.^17^ However, our findings indicate that voluntary LLM use may offer a way to address this trade-off, as it appears to mitigate the reduction in perceived information processing while maintaining low levels of perceived role overload. Thus, the effects of LLM use on work design seem to vary across different work characteristics and between instructed or voluntary LLM use.

Consistent with Parker and Grote's^10^ theoretical framework, we identified voluntariness as a relevant higher-order factor for the effects of technology on work design, mitigating negative effects of LLM use. Aligning with more recent theoretical models^13,27^ that outline the importance of human control, we demonstrate that voluntary LLM use can enrich work design by mitigating undesired yet maintaining desired work design consequences. Thereby, we provide causal evidence for the theoretically proposed but rarely tested role of voluntariness in moderating AI's effects on work design and employee outcomes. Prior experimental research [e.g.,^3,17^] has primarily focused on the comparison of LLM use and non-use. An essential contribution of our study lies in demonstrating the importance of voluntary LLM use as a third and seemingly more benficial usage mode, thereby positioning voluntariness as a key factor for future models.

Supporting the transferability of effects found for other technologies at work, our results suggest that voluntary LLM use affects work design comparable to adaptable automation regarding certain work characteristics. Consistent with Rieth et al.,^15^ who found positive effects of adaptable automation on perceived autonomy without impairing workload or performance, we find similar effects for voluntary LLM use. Our study contributes to prior findings by extending the context of automation to a human-in-the-loop approach and investigating information processing as an additional work characteristic that benefits from flexible technology integration.

Further aligning with Parker and Grote,^10^ we identified need for autonomy as an individual factor moderating the effects of technologies on work design: a high need for autonomy seems to amplify the effects on perceived decision-making autonomy. However, need for cognition did not moderate the effects on perceived information processing in our study, suggesting no essential role of cognitive engagement in our setting. In contrast, on a descriptive level we found negative correlations between need for cognition and both perceived information processing and role overload (Table 1), indicating that a higher need for cognition is associated with lower perceived cognitive demands.

Furthermore, consistent with Hickman and Langer's^27^ conceptual model, we found that (voluntary) LLM use influences an indicator of job performance in a decision-making task. While Vaccaro et al.^11^ demonstrated the potential to improve decision-making performance in human-AI collaboration, we provide causal evidence that voluntary AI use serves as a key factor in achieving such improvements.

Overall, our study contributes to the existing body of literature by providing causal evidence for theoretical frameworks [e.g.,^10,13,27^] and by extending prior empirical evidence [e.g.,^3,11,15,17^]. Especially the role of voluntary LLM use and individual needs represent a novel perspective that may enrich existing theories and guide future research on AI-integration in organizations.

### Practical implications

Our results offer critical insights for organizational practice by providing empirical evidence on the causal effects of instructed and voluntary LLM use on work characteristics and decision-making performance. While instructed LLM use seems to reduce perceived decision-making autonomy, perceived information processing, and performance – thereby deteriorating job quality and employee outcomes – it also yields positive effects, such as reduced role overload. For practitioners, these findings underscore the need to carefully consider the negative impacts on autonomy, information processing, and performance when integrating LLMs into work processes.

More importantly, our results highlight a promising organizational factor to mitigate negative impacts while also reaping the benefits of LLM use: voluntariness. Across all dependent variables, our results stress that organizations should refrain from mandatory LLM use and instead empower employees to adopt LLMs voluntarily. This approach ensures that decision-making autonomy, information processing, and performance remain robust while still reducing role overload. In contrast, mandatory LLM use may additionally have negative consequences for employees’ sense of responsibility, motivation, psychological safety and well-being, potentially undermining important sources of work meaningfulness.^27,47^

Moreover, our moderating analyses show that voluntariness is particularly important for employees with a high need for autonomy. Therefore, organizations should also take individual factors into account, as they might make the difference in positive and negative effects of LLM use at work.

### Limitations and future research

Besides the important theoretical and practical implications of our findings, we have to acknowledge several limitations of our study. First, given that the type of technology is supposed to influence its effects on work design,^12,18^ it remains unclear whether our findings can be transferred to working with other technologies in terms of AI or LLM models. We therefore emphasize the need to replicate our hypotheses with other technologies, such as intelligent assistance systems or other AI-based systems.

Second, in the current study we focused on a subset of work characteristics outlined in various work design frameworks, such as the SMART model of work design.^18^ By investigating decision-making autonomy, information processing, and role overload, we cover work characteristics which are, according to the SMART model, subordinate to stimulating, autonomous, and tolerable work characteristics.^18^ To gain a more holistic understanding of how LLM use affects employees through altered work characteristics, we emphasize the importance of also investigating other work characteristics, such as mastery (e.g., feedback from the job) and relational work characteristics (e.g., social support).

Third, as individual factors we only investigated the potential role of need for autonomy and cognition. While we identified need for autonomy as a crucial moderating factor, we did not find support for the moderating role of need for cognition. Considering Van den Broeck et al.'s^48^ review of basic psychological needs at work, need for competence and need for relatedness could also represent relevant factors in the context of LLM use at work. Future research should also investigate the role of satisfaction of psychological needs besides need strength – as such a need operationalization could essentially mediate the effects of work characteristics on employee outcomes, such as performance, health, or job satisfaction.^12,27,49^

Fourth, we deployed a relatively short online-experiment in which participants executed two simulated work tasks. Therefore, it remains unclear how LLM use alters work characteristics, the satisfaction of psychological needs and eventually, performance outcomes in real work settings and in the long run. Furthermore, the tasks used in this study represent only a limited subset of workplace activities and may not fully reflect how employees interact with LLMs in their everyday work. Future research should also examine whether the observed effects generalize across industries, occupational groups, work arrangements (e.g., full-time, part-time, and hybrid work), cultural contexts, and different LLM systems. To address these limitations, longitudinal studies are needed to increase external validity and gain a better understanding of how instructed and voluntary LLM use shape employee outcomes mediated by altered work design in organizational practice.^27^

## Conclusion

Overall, our findings suggest that instructed or mandatory LLM use may negatively affect important work characteristics, including perceived decision-making autonomy and information processing, and may also impair decision-making performance, despite reducing perceived role overload. However, our results indicate that voluntariness can mitigate these adverse effects while preserving the benefits of LLM use, such as reduced role overload. Especially individuals with a high need for autonomy seem to perceive instructed LLM use as particularly restrictive, while perceiving voluntary LLM use as particularly liberating in regards to decision-making autonomy. Our experiment provides essential contributions for the implementation of LLMs that benefits work design and, ultimately, employee outcomes. In particular, we identify voluntariness of LLM use as a key factor for both theory and practice and highlight the importance of integrating individual factors into research and organizational approaches to AI adoption.

## Data Availability

The data that support the findings of this study are openly available in the Open Science Framework (OSF) at https://osf.io/fj938.
